# Large-scale identification and evolutionary analysis of ATP-binding cassette transporters in cross-lineage plant species

**DOI:** 10.1093/hr/uhag209

**Published:** 2026-06-01

**Authors:** Kangkang Song, Yinuo Zhao, He Xu, Haozhen Li, Xiaohua Zhang, Yanru Song, Boyuan Liu, Jingyu Wang, Bin Tan, Zhe Zhang, Bin Li, Wenjing Liu, Yiying Qi, Long Yang

**Affiliations:** College of Plant Protection and Agricultural Big-Data Research Center, Shandong Agricultural University, Taian 271018, China; College of Plant Protection and Agricultural Big-Data Research Center, Shandong Agricultural University, Taian 271018, China; College of Plant Protection and Agricultural Big-Data Research Center, Shandong Agricultural University, Taian 271018, China; College of Plant Protection and Agricultural Big-Data Research Center, Shandong Agricultural University, Taian 271018, China; College of Plant Protection and Agricultural Big-Data Research Center, Shandong Agricultural University, Taian 271018, China; College of Plant Protection and Agricultural Big-Data Research Center, Shandong Agricultural University, Taian 271018, China; College of Plant Protection and Agricultural Big-Data Research Center, Shandong Agricultural University, Taian 271018, China; College of Plant Protection and Agricultural Big-Data Research Center, Shandong Agricultural University, Taian 271018, China; College of Plant Protection and Agricultural Big-Data Research Center, Shandong Agricultural University, Taian 271018, China; School of Materials Science and Engineering, Shanghai Institute of Technology, Shanghai 201418, China; The Key Laboratory of Plant Development and Environmental Adaptation Biology, Ministry of Education, School of Life Sciences, Shandong University, Qingdao 266237, China; College of Plant Protection and Agricultural Big-Data Research Center, Shandong Agricultural University, Taian 271018, China; College of Plant Protection and Agricultural Big-Data Research Center, Shandong Agricultural University, Taian 271018, China; College of Plant Protection and Agricultural Big-Data Research Center, Shandong Agricultural University, Taian 271018, China

## Abstract

The ATP-binding cassette (ABC) gene family plays a crucial role in plants by mediating the transport of various molecules, contributing to development, stress responses, and secondary metabolism. Here, 159077ABC genes were identified from 1108 plant species. Comparative analysis revealed that higher plants had more ABC genes compared to algae plants. The ABCG1, ABCG4, and ABCG8 subfamilies originated in land plants and subsequently underwent distinct evolutionary pathways. Motif 1 and motif 9 demonstrated a conserved high-frequency pattern across different lineages. The expansion of ABC family mainly occurred through dispersed and whole-genome duplication in higher angiosperms, and full-length transposons may mediate the neofunctionalization of these genes. The pan-ABC family analysis of 12 tomato accessions revealed more relaxed selective pressures in dispensable ABC genes. The ABC genes of *Arabidopsis thaliana* exhibited stronger responsiveness and connectivity degree under salt stress. To facilitate further research, we established the PlantABC database (http://biodb.com.cn/PlantABC/index.html), which contains comprehensive genomic and evolutionary information about plant ABC genes. This study provides valuable perspective and resources for advancing research on the evolution and functions of plant ABC genes.

## Introduction

Plants require a complex array of physiological processes during growth and development, many of which depend on the precise transport and metabolism of essential compounds such as auxins, gibberellins, and lipids [1, 2]. In addition to developmental regulation, plants must cope with increasing abiotic stresses including heat stress, salt stress, alkaline stress, and heavy metal toxicity [1, 3]. These complex environmental conditions disrupt normal plant physiological processes and pose serious threats to agricultural productivity and food security [4]. ATP-binding cassette (ABC) transporters, one of the largest transporter families, play extensive and crucial roles in substance transport, organ development, stress response, and heavy metal detoxification [5–7]. However, there remains a lack of comprehensive understanding regarding the molecular characteristics and evolutionary features of ABC gene families in plants.

Research on plant ABC transporters began with the identification of *AtABCB1* in *Arabidopsis thaliana* [8], where a total of 129 ABC genes were later classified into eight subfamilies (ABCA–ABCI) [9]. Among these, the ABCB, ABCC, and ABCG subfamilies have been most extensively characterized [10–13]. Functional ABC transporters consisted of two functional domains: the nucleotide-binding domain (NBD) that hydrolyzes ATP to provide energy and the transmembrane domain (TMD) that facilitates substrate recognition and translocation [14]. The NBD domain is relatively conserved across different species and contains Walker A, Walker B, and the ABC signature motifs (also known as Walker C) [15]. Walker A and Walker B motifs were conserved across all ATP-binding proteins, whereas Walker C was unique to ABC transporters. In contrast, the TMD domain exhibits high sequence variability and often contains multiple transmembrane helices. ABC transporters accommodated a wide range of substrates, including inorganic ions (e.g. metals), small organic molecules (e.g. sugars and amino acids), and macromolecules (e.g. peptides and lipids) [16]. This broad substrate spectrum endowed ABC transporters with critical roles in cellular homeostasis, environmental stress responses, and secondary metabolism regulation [17, 18].

Functional studies have demonstrated that the plant ABC gene family played a crucial role in various physiological processes. For instance, they mediate phytohormone transport, as exemplified by *AtABCB19* in brassinosteroids efflux in *Arabidopsis* [19]. They are also key players in secondary metabolite accumulation, such as *ABCC8* contributed to anthocyanin accumulation in strawberry fruits [20]. Beyond these roles, ABC transporters participate in reproductive development—including female gametophyte and seed development in rice (*OsABCB24*) [21] and stigma exsertion in *Medicago truncatula* (ABCG proteins) [22]. In bitter gourd, the ABCG gene *Moc03g21510* was identified as the best candidate gene highly associated with pedicel elongation [23]. Additionally, they contribute to functional cuticle formation [24], lignin accumulation [25], shoot branching [26], blue light responses [27], and grain filling [28]. ABC genes also regulated plant stress tolerance, including cadmium stress [29], aluminum stress [30], glyphosate resistance [31], drought stress [32], and biotic stress [33, 34].

Despite these advances, the ABC gene repertoire in many plants, especially horticultural plants and crops, remain uncharacterized. The molecular characteristics, evolutionary mechanisms, and regulatory networks of ABC gene families across different plant groups need to be systematically analyzed. With the rapid advances in sequencing technologies and bioinformatics, the assembly of numerous plant genomes and the updating of specialized omics databases provide advantageous conditions for the analysis of the ABC gene family across the plant kingdom [35–37]. In this study, the ABC gene family of 1108 plant species was comprehensively identified and classified. We systematically investigated the evolutionary trajectories and mechanisms of the ABC gene family in representative species and conducted a pan-genome analysis of ABC genes in 12 tomato accessions to explore their evolutionary patterns and potential functions. Furthermore, we established a plant ABC gene family database to serve as a central resource for the research community. These studies provide comprehensive insights into the evolution and functional diversification of ABC transporters in plants and facilitates future efforts in crop improvement.

## Results

### ABC gene family in 1108 plant species

A total of 159077ABC genes were identified across 1108 plant species encompassing eight lineages, ranging from algae to eudicots. A comparative analysis revealed that the number of ABC family members in land lineages was significantly higher than algae (Fig. 1A and Table S1). This expansion was particularly prominent across the ABCB, ABCC, and ABCG subfamilies (Fig. 1C, D, and  H). Compared with algae, angiosperms exhibited a similar but statistically nonsignificant increase in ABCI gene number (Fig. 1I). In addition, the gene numbers in the ABCA, ABCD, ABCE, and ABCF subfamilies exhibited extensive variation across different plant lineages (Fig. 1B, E, F, and G).

**Figure 1:**
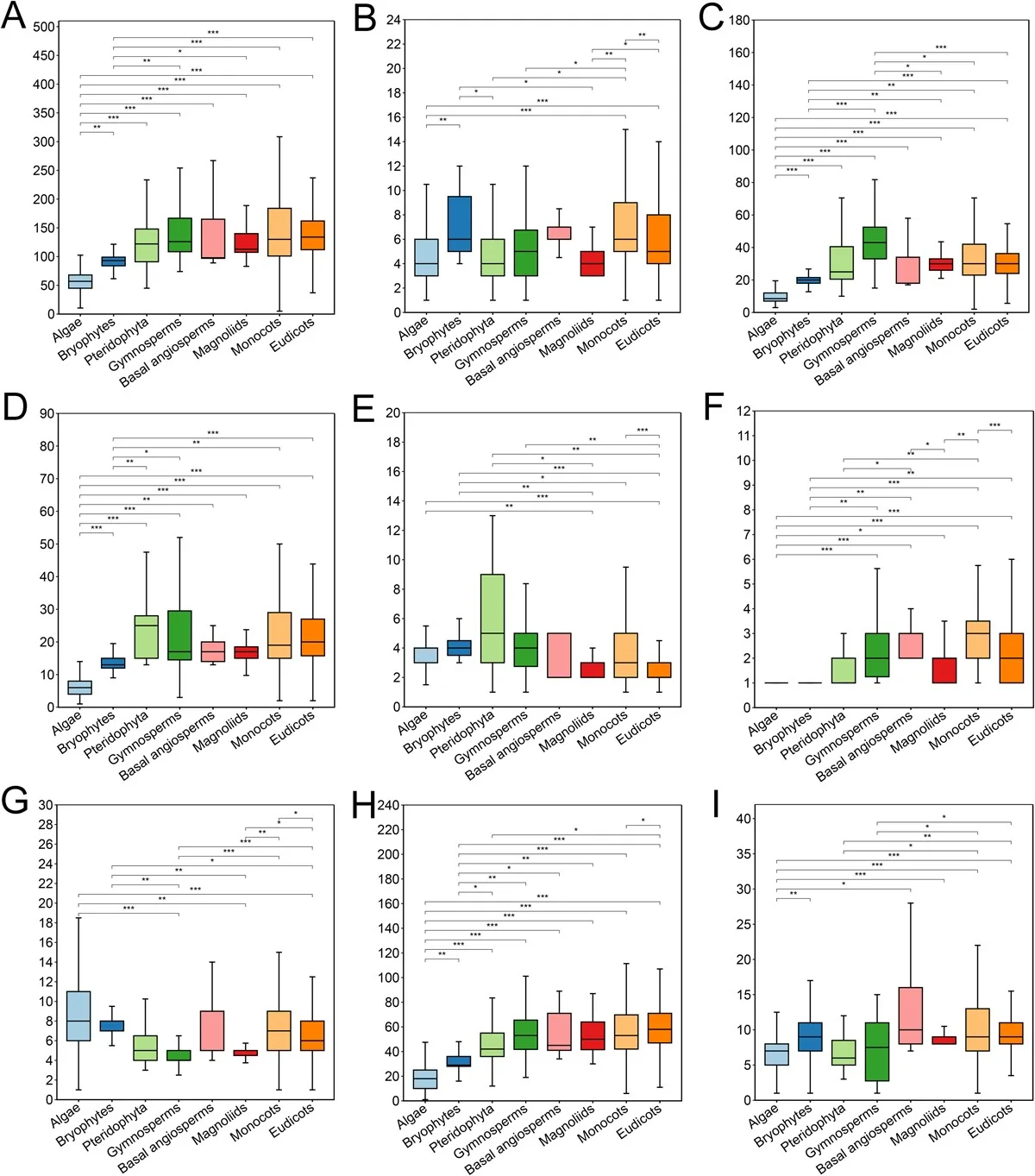
The gene number of ABC family in eight lineages. (A) The gene number of all ABC subfamily in eight lineages. (B–I) The gene number of each ABC subfamily (ABCA, ABCB, ABCC, ABCD, ABCE, ABCF, ABCG, and ABCI) in eight lineages.

The investigation of protein length distribution revealed evolutionary trends in ABC protein structure (1). ABC proteins in bryophytes and pteridophyta plants were longer than those in algae plants. Furthermore, the ABC proteins in basal angiosperms, magnoliids, monocots, and eudicots plants contained more amino acid residues than those in gymnosperms. Notably, the length of ABC proteins longer than 1000 amino acids exhibited a single peak in basal angiosperms and earlier-evolved plants, while monocots and eudicots display a distinct bimodal pattern (Fig. S1). GO enrichment analysis indicated that proteins contained in the above peak played a role in transcriptional regulation, genome maintenance, and hormone signaling transduction, whereas the proteins contained in the below peak were more involved in physiological processes such as catabolism, primary metabolism, and membrane transport (Fig. S2).

### Phylogenetic reconstruction of the ABC gene family

To elucidate the evolutionary history of the ABC gene family, a phylogenetic tree using 1024 ABC genes from nine representative species spanning major plant lineages was constructed (Fig. 2A). *Physcomitrium patens* had the largest number of ABC family genes (149), followed by *Selaginella moellendorffii* (136), *A. thaliana* (127), and *Solanum lycopersicum* (121). The lowest number was found in the green alga *Chlamydomonas reinhardtii*, with 86 ABC family genes. Phylogenetic analysis showed that all ABC family genes could be classified into eight groups, defined as ABCA–ABCG and ABCI subfamilies (Fig. 2A). Four hundred five, 219, and 153 ABC family genes were found in the ABCG, ABCB, and ABCC subfamilies, respectively. The number of genes in the other five subfamilies was all fewer than 100, with the ABCE subfamily having the least, containing only 19 genes. The ABCB, ABCC, and ABCG subfamilies exhibited convergent expansion across all plant lineages. Homologous genes within the same species were clustered together, indicating the lineage specificity of the ABC gene family (Fig. 2A).

**Figure 2:**
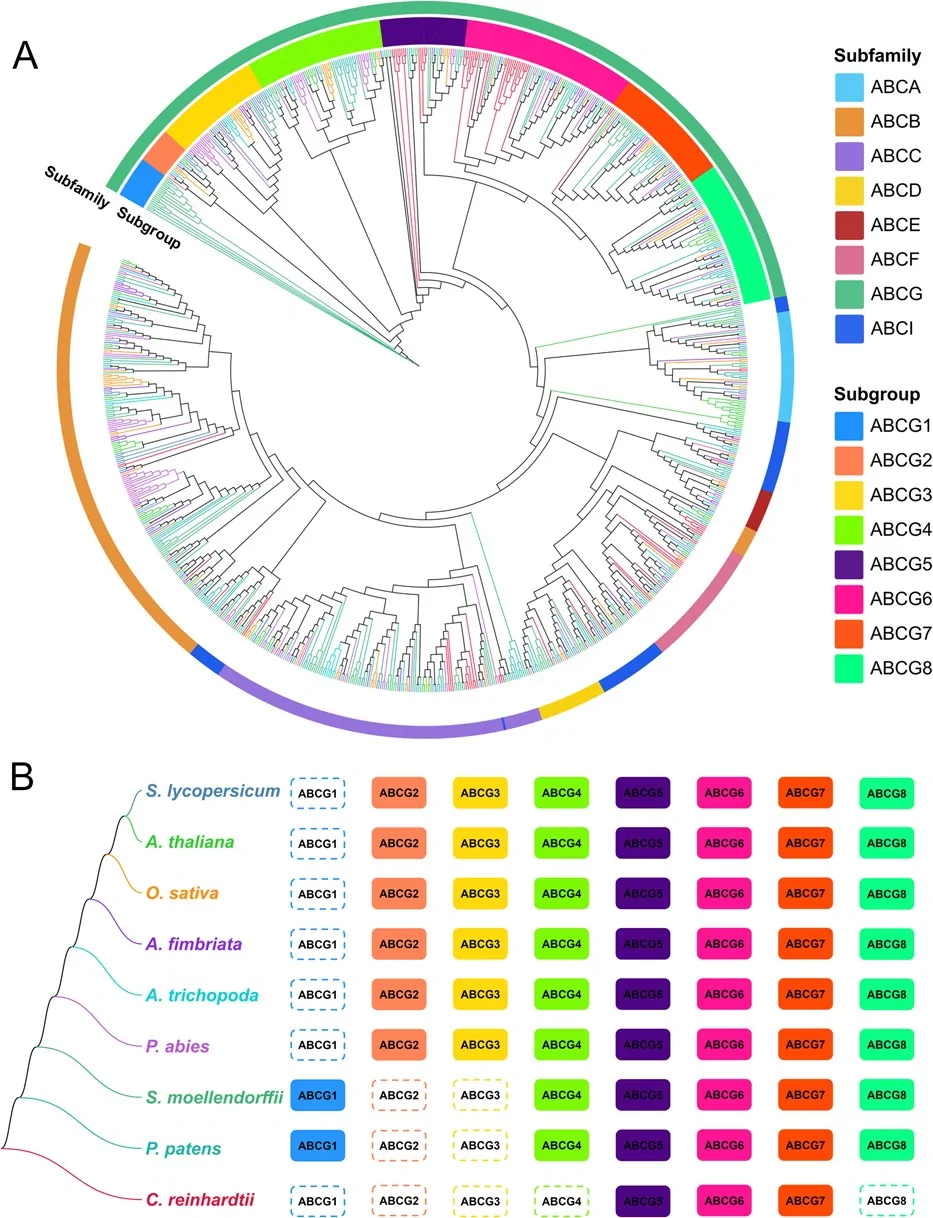
Phylogenetic and evolutionary trajectory of ABC gene family. (A) Phylogenetic tree of 1024 ABC genes from nine representative species. The branch colors represent different species, as indicated in panel (B). The outer circle colors represent different subfamilies, and the inner circle colors represent different subgroups of the ABCG subfamily. (B) Evolutionary trajectory of the ABCG subfamily from lower algae to higher eudicot representative plants. Boxes colors represent distinct subgroups. Solid boxes indicate present subgroups, and dashed boxes denote absent subgroups.

Based on phylogenetic relationships, the ABCG subfamily was divided into eight subgroups (Fig. 2A). These subgroups exhibited distinct evolutionary trajectories across representative species of different lineages (Fig. 2B). The ABCG1, ABCG4, and ABCG8 subgroups first emerged in the bryophytes *P. patens* but underwent different evolutionary pathways. ABCG1 was retained in the pteridophyta *S. moellendorffii* but was lost in subsequent evolution. ABCG4 and ABCG8 persisted and were retained in later evolution within higher plant lineages. ABCG1, ABCG4, and ABCG8 may have played active roles in plant terrestrial adaptation. The ABCG2 and ABCG3 subgroups first appeared in the gymnosperms *P. abies* and were consistently retained throughout evolution, potentially associated with environmental adaptation in seed plants. ABCG5, ABCG6, and ABCG7 emerged early in the algae *C. reinhardtii* and were continuously retained in land plants, suggesting fundamental roles in plant growth and development.

### Conserved motif evolution of ABC gene family

Ten motifs were identified in the 1024 ABC protein sequences from the nine representative species (Fig. 3). Each lineage species contained all 10 motifs, emphasizing the structural conservation of the ABC family across different plant groups. Motif 3 was the longest, followed by motif 10 and motif 5, while motif 2 was the shortest (Fig. S3). Motif 1 and motif 9 showed the highest frequency (Fig. 3A), suggesting their fundamental functional importance. Compared to *A. thaliana*, the frequency of nearly all 10 motifs, including motif 1 and motif 9, increased in *Solanum lycopersicum*.

**Figure 3:**
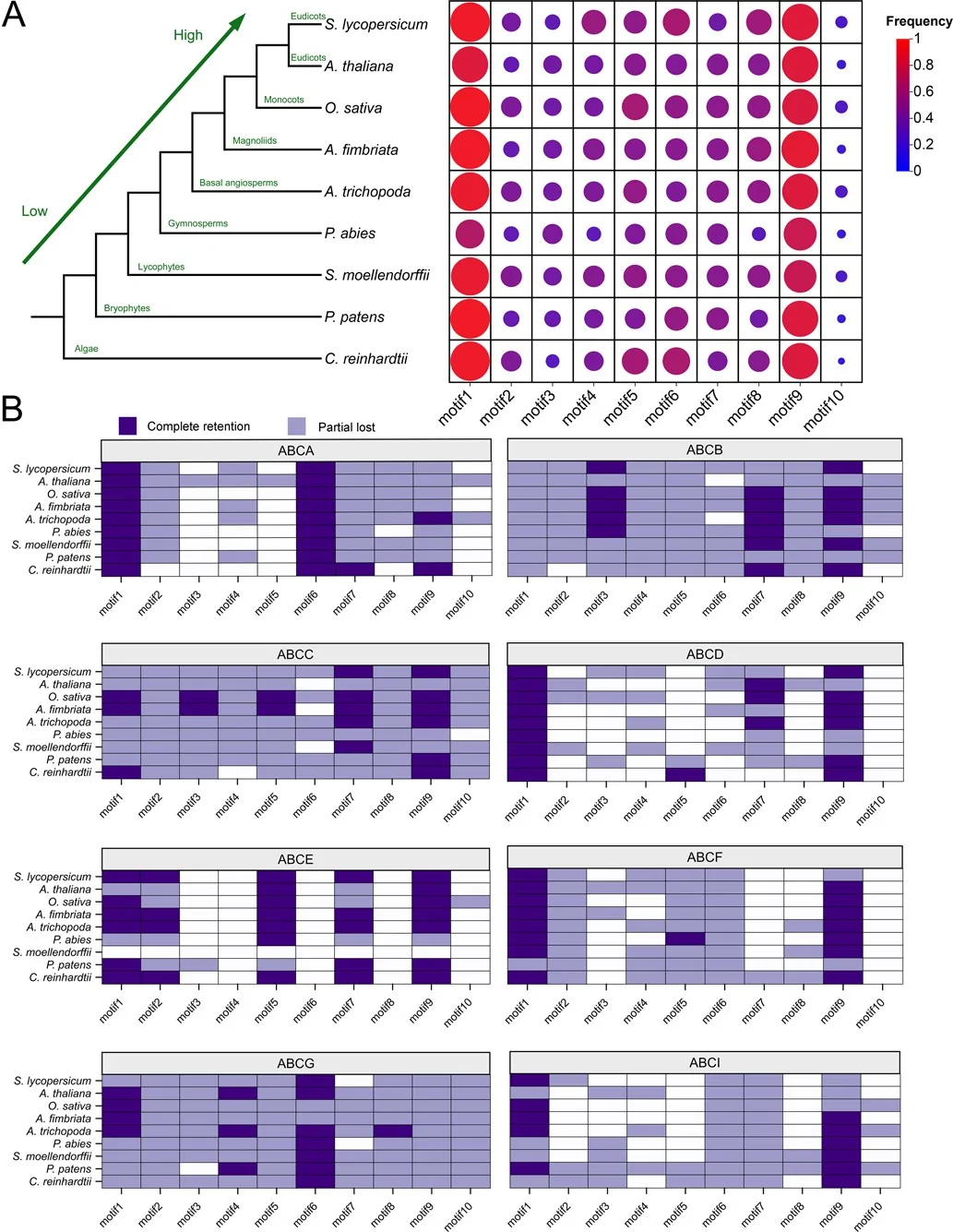
Motif evolution ABC gene family of nine representative species. (A) Motif frequency of nine representative species. (B) Motif evolutionary trajectory of in ABC subfamily. Deep purple represents complete retention, light purple represents partial loss, and white represents complete loss.

The evolutionary trajectory of motifs in the ABC gene family was revealed (Fig. 3B). The ABCB, ABCC, and ABCG subfamilies contained the most abundant motifs. In other subfamilies, motifs exhibited multiple independent events of gain and loss across divergent lineages. Motif 1 was completely retained in ABCA and ABCD subfamily throughout all lineage species. Motif 10 was partially lost in the ABCG subfamily across all lineage species and was entirely absent in the ABCD and ABCF subfamilies (Fig. 3B).

### Duplication pattern of ABC gene family

Substantial genomic turnover of ABC genes was observed across major plant lineages, characterized by both lineage-specific duplications and losses (Fig. 4A and B). An analysis of ancestral nodes revealed distinct patterns of gene family dynamics. In the common ancestor of land plants and the common ancestor of angiosperms, unlike other ancestral lineages, the number of duplications of ABC genes was more than gene losses. Specifically, the common ancestor of land plants experienced a notable expansion, with 96 gene duplications and 17 gene losses. Similarly, the ancestor of *A. trichopoda* and higher angiosperms species occurred 70 gene duplication events and 27 gene losses. In contrast, along the evolutionary path from the algae *C. reinhardtii* to the monocot *O. sativa*, gene losses consistently exceeded duplications. Expansion of the ABC family resumed only in later-diverging eudicot lineages, as exemplified by *A. thaliana* and *S. lycopersicum*, where duplications again surpassed losses (Fig. 4A and B).

**Figure 4:**
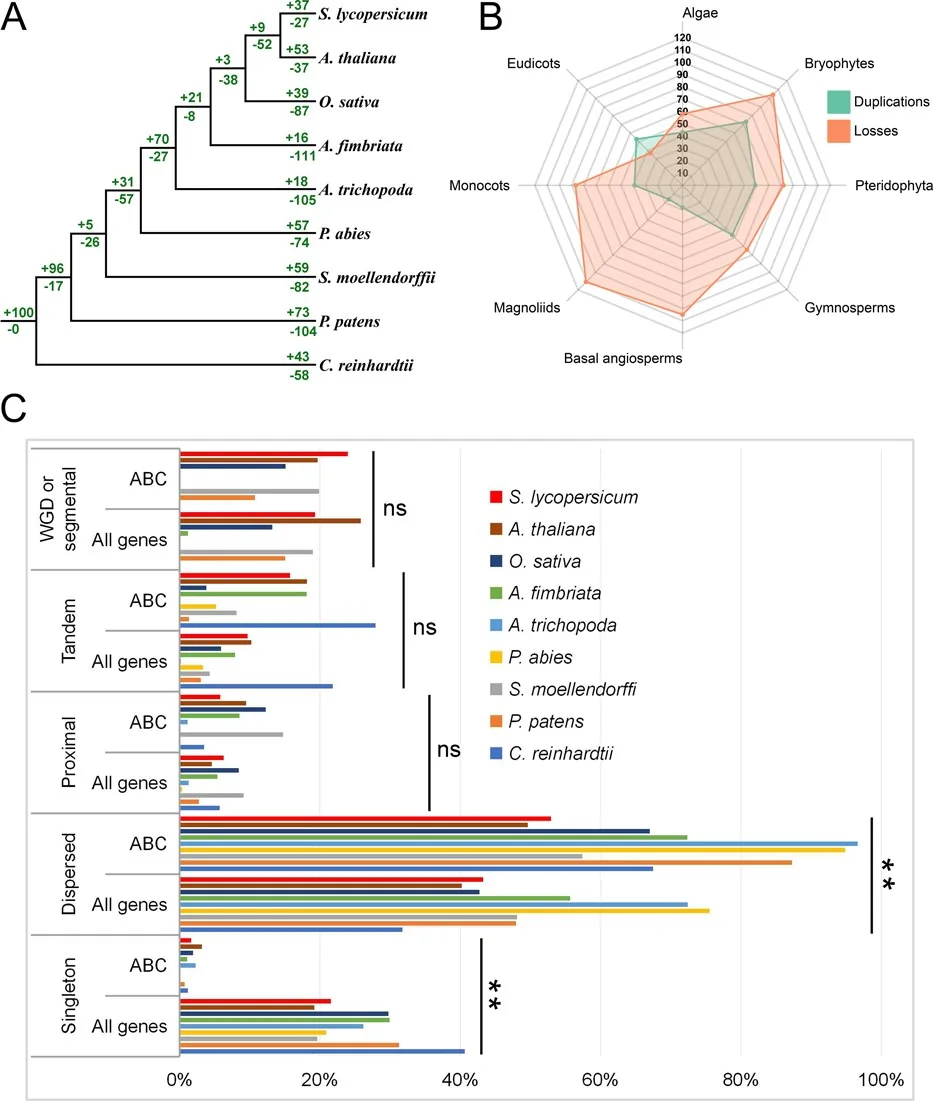
Duplication pattern of ABC gene family of representative species. (A, B) Duplication and loss of nine representative species. (C) Duplication types of nine representative species. Significant differences in five duplication types between ABC genes and all genes were examined using the Wilcoxon test.

Five duplication types of the ABC gene family were identified, including singleton, dispersed, proximal, tandem, and whole-genome duplication (WGD) (Fig. 4C). Compared to the genomic background, ABC genes exhibited a significantly lower proportion of singleton duplication but a markedly higher contribution from dispersed duplication (Fig. 4C). This trend was particularly pronounced in the basal angiosperm *A. trichopoda*, where 96.63% of ABC genes arose from dispersed duplication. WGD has been considered as an important genetic driver to angiosperm adaptation to environmental changes. Notably, 15.09% of ABC genes in *O. sativa*, 19.69% in *A. thaliana*, and 23.97% in *S. lycopersicum* were expanded through WGD (Fig. 4C). These results indicated that large-scale duplication mechanisms played an important role in the expansion of ABC genes, accompanied by chromosome doubling, particularly in angiosperms.

### Collinearity and selection pressure of ABC gene family

Given the distinct WGD tendency observed in the angiosperms *O. sativa*, *A. thaliana*, and *S. lycopersicum*, we further investigated the collinearity and selection pressure of ABC genes in these three species. The eight paralogous gene pairs were found in *O. sativa* (Fig. 5A)*,* while 13 and 18 paralogous gene pairs were identified in *A. thaliana* and *S. lycopersicum* (Fig. 5B and C). A total of 55 orthologous gene pairs were found between *S. lycopersicum* and *A. thaliana* (Fig. 5D). The *A. thaliana* ABCG14 gene expanded into two genes in *S. lycopersicum*, namely, *Solyc08g075430.3* and *Solyc08g005580.3* (Fig. 5D). Thirteen orthologous gene pairs were identified between *S. lycopersicum* and *O. sativa* (Fig. 5E).

**Figure 5:**
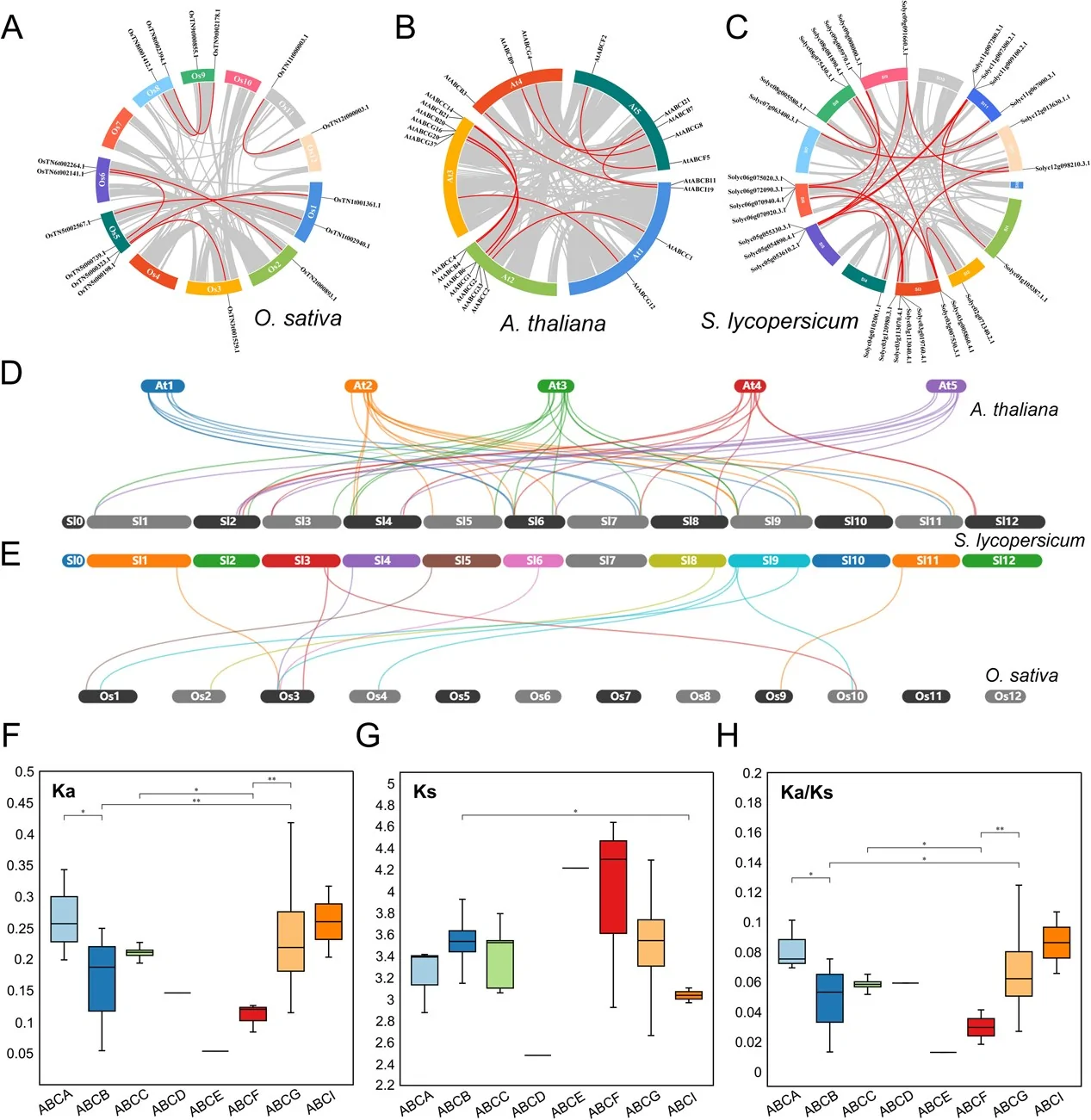
Collinearity and selection pressure of ABC gene family in representative angiosperms. (A) Intraspecific synteny analysis of the ABC gene family in rice. (B) Intraspecific synteny analysis of the ABC gene family in tomato. (C) Intraspecific synteny analysis of the ABC gene family in *Arabidopsis*. (D) Interspecific collinearity of ABC gene family in tomato and *Arabidopsis*. (E) Interspecific collinearity of ABC gene family in tomato and rice. (F) Ka values of orthologous genes in *O. sativa*, *A. thaliana,* and *S. lycopersicum*. (G) Ks values of orthologous genes in *O. sativa*, *A. thaliana,* and *S. lycopersicum*. (H) Ka/Ks values of orthologous genes in *O. sativa*, *A. thaliana,* and *S. lycopersicum*.

Following the identification of extensive duplication events, we sought to understand the evolutionary forces that have shaped the ABC gene family after divergence. To this end, we calculated the Ka, Ks, and Ka/Ks values for orthologous ABC genes in *S. lycopersicum*, *A. thaliana*, and *O. sativa*. The Ka values showed more significant differences between subfamilies compared to Ks (Fig. 5F and G). The Ka/Ks values for all subfamilies were <1, highlighting that all ABC genes have evolved under predominant purifying selection (Fig. 5H). The strength of this constraint, however, varied across subfamilies. The ABCE subfamily experienced the strongest purifying selection, as reflected by the lowest Ka/Ks value. Subfamilies such as ABCC and ABCF displayed moderate constraint, while subfamilies ABCB and ABCG showed broader distributions of Ka/Ks values, suggesting greater variability in selective pressures and potential functional differentiation within the subfamily genes (Fig. 5H).

### Transposable element characteristics in ABC genes and flanking regions

To explore the potential role of transposable element (TE) in shaping the evolutionary dynamics of ABC gene family, we investigated TE insertions within the gene body, as well as the 2 kb upstream and downstream regions in *A. thaliana*, *O. sativa,* and *S. lycopersicum*. A total of 11 types of TEs were identified, including seven DNA transposons and four retrotransposons (Fig. 6A–C). DNA/TcMar transposons had the highest insertion ratio in gene body and upstream regions, whereas DNA/PIF-Harbinger transposons showed the highest insertion densities in downstream regions (Fig. 6A–C). Notably, the ABCB, ABCF, and ABCG subfamilies, the largest and most expanded subfamilies, exhibited a greater abundance of TE insertions, particularly in upstream regions (Fig. 6D).

**Figure 6:**
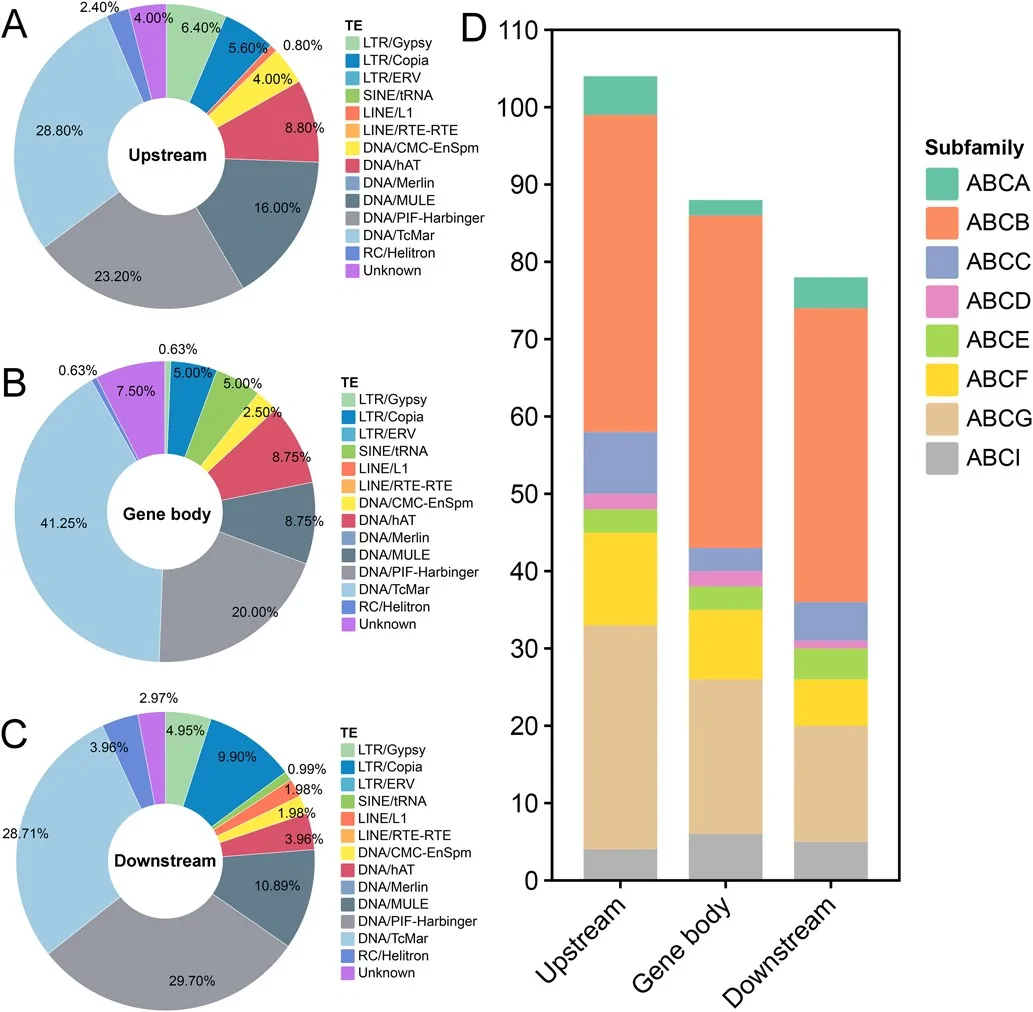
TE insertions of ABC gene family in representative angiosperms. (A) Transposable element statistics of gene body region. (B) Transposable element statistics of upstream 2000 bp region. (C) Transposable element statistics of downstream 2000 bp region. (D) Distribution of the ABC Subfamily of transposable element.

### Comprehensive profiling and copy number variation of tomato Pan-ABC family

Through the aforementioned research, we have revealed that tomato ABC genes exhibit higher motif frequency, more pronounced WGD duplication events and broader collinearity patterns. However, variations in the ABC family among different tomato germplasms remain unclear. We selected 12 tomato germplasms for a pan-genome analysis of the tomato ABC gene family, including one cultivated large-fruited tomato (*S. lycopersicum*), one cherry tomato (*S. lycopersicum* var. *cerasiforme*, an intermediate type between wild ancestors and modern cultivated varieties), and 10 wild tomato accessions (Table S2).

The 1547 ABC genes were identified in 12 tomato accessions, with an average of 129 genes per germplasm. One hundred and twenty-one ABC genes were found in *S. lycopersicum*, and a large number of ABC genes were also uncovered in *S. lycopersicum* var. *cerasiforme* (125), *Solanum chilense* (140), *Solanum chmielewskii* (122), *Solanum corneliomulleri* (129), *Solanum galapagense* (126), *Solanum habrochaites* (120), *Solanum lycopersicoides* (149), *Solanum neorickii* (128), *Solanum pennellii* (131), *Solanum peruvianum* (128), and *Solanum pimpinellifolium* (128).

All tomato ABC genes were classified into 122 orthologous gene groups (OGGs) (Fig. 7A). The ABC genes of *S. lycopersicum* were only assigned to 107 OGGs. Based on the number of accessions included in each OGG, 122 ABC OGGs were categorized into 79 core ABC OGGs, 25 softcore ABC OGGs, 12 dispensable ABC OGGs, and 6 private ABC OGGs (Table S3), accounting for 64.75%, 20.49%, 9.84%, and 4.92% of all ABC OGGs, respectively (Fig. 7B). Several core OGGs showed a large number of copies across all tomato accessions, such as OG0000000, OG0000002, OG0000003, OG0000004, and OG0000005 (Fig. 7C). There was considerable variation in gene copy numbers within OGGs between different tomato accessions (Fig. 7C). For example, in OG0000012 (core ABC OGGs), *S. lycopersicum* had one ABC gene, while *S. lycopersicoides* had four ABC genes. In OG0000001 (softcore ABC OGGs), both *S. lycopersicum* and *S. pennellii* had four ABC copies, while *Solanum habrochaites* lacked ABC genes.

**Figure 7:**
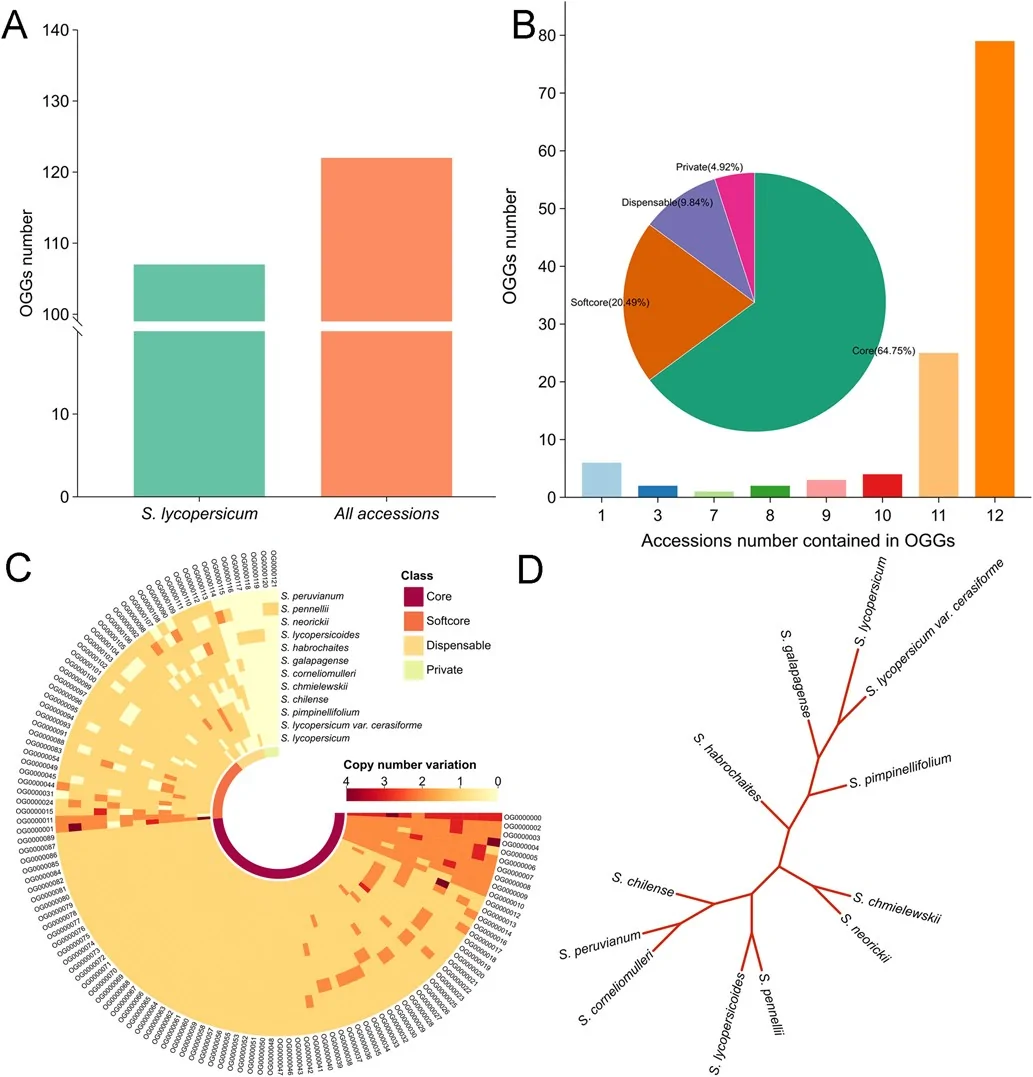
The overview of the tomato Pan-ABC family. (A) OGG number of *S. lycopersicum* and all accessions. (B) OGGs number contained specific accessions. (C) The copy number variation of ABC gene in tomato among different accessions. (D) Phylogenetic tree based on tomato ABC single copy gene.

The phylogenetic tree constructed from ABC single-copy genes across 12 tomato species revealed distinct evolutionary relationships among the analyzed taxa (Fig. 7D). We compared the ABC gene tree with the well-established species tree. *Solanum lycopersicum* was closest in evolutionary relationship to *S. lycopersicum* var. *cerasiforme*, consistent with the species tree topology, suggesting conserved ABC gene evolution in domesticated species. *S. pennellii* and *Solanum habrochaites* were positioned on separate branches, deviating from the species tree topology, indicating potential adaptive divergence in ABC gene function.

### Selective pressure and functional innovation in tomato Pan-ABC family evolution

Core ABC OGGs exhibited lower Ka and higher Ks compared to softcore ABC OGGs，suggesting that core ABC OGGs may be under stronger purifying selection to maintain essential biological functions (Fig. 8A and B). A progressive increase in Ka/Ks ratios was observed from core ABC OGGs to softcore ABC OGGs to dispensable ABC OGGs, with significant differences between each category (Fig. 8C). This pattern suggests that dispensable genes evolve under more relaxed selective constraints and might even be experiencing episodes of positive selection, driving functional innovation and adaptation to specific environmental niches.

**Figure 8:**
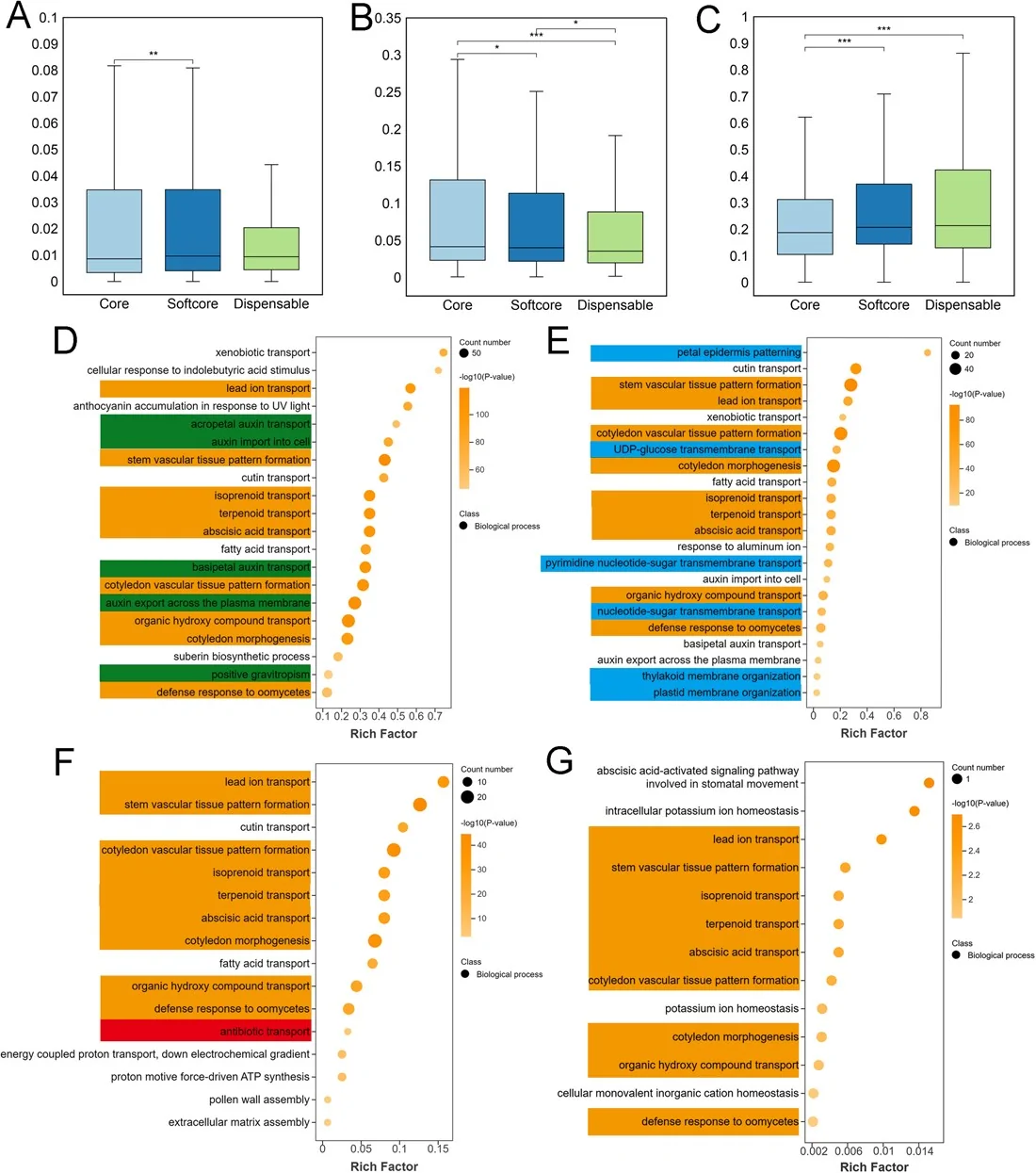
Selection pressure and GO enrichment in tomato pan-ABC family. (A) Ka of core, softcore, and dispensable ABC OGGs. (B) Ks of core, softcore, and dispensable ABC OGGs. (C) Ks/Ks of core, softcore, and dispensable ABC OGGs. (D) Top 20 most significant GO enrichment of core ABC OGGs. (E) GO enrichment of softcore ABC OGGs. (F) GO enrichment of dispensable ABC OGGs. (G) GO enrichment of private ABC OGGs.

Functional enrichment analysis further highlighted the distinct roles of these OGG categories. The core ABC OGGs were significantly enriched for 78 Gene Ontology (GO) terms, encompassing key biological processes such as the regulation of metabolic processes (e.g. phenylpropanoids, glycosyl compounds) and stress defense responses (e.g. heavy metal ion homeostasis, pathogen defense), as well as cell structure formation (e.g. cell wall assembly, organ boundary specification) (Fig. 8D and Table S4). Notably, the core ABC OGGs showed significant enrichment in functions related to auxin transport, including auxin export across the plasma membrane, basipetal auxin transport, acropetal auxin transport, auxin import into the cell, and positive gravitropism (Fig. 8D). The softcore ABC OGGs possessed 22 enriched terms, including six unique functions related to sugar transport (UDP–glucose transmembrane transport, pyrimidine nucleotide–sugar transmembrane transport, nucleotide–sugar transmembrane transport), membrane formation (plastid membrane organization, thylakoid membrane organization), and reproductive growth (petal epidermis patterning) (Fig. 8E). Dispensable OGGs were uniquely enriched in antibiotic transport, a function might facilitate the removal of exogenous antimicrobial agents derived from organic fertilizers and pesticides (Fig. 8F). Private OGGs shared some functional terms with core OGGs (Fig. 8G), which may indicate the neofunctionalization of existing important biological pathways in specific lineages.

### Divergent expression patterns of ABC genes across plant lineages

To investigate the differential stress responses of ABC genes in lower and higher plants, the expression patterns of ABC genes under salt stress were analyzed in *C. reinhardtii* and *A. thaliana*. A total of 38 ABC genes were found to be differentially expressed in both *C. reinhardtii* and *A. thaliana* (Fig. 9A). Compared with *C. reinhardtii*, *A. thaliana* exhibited greater fold changes in the expression of ABC genes under salt stress and possessed a higher number of up-regulated ABC genes.

**Figure 9:**
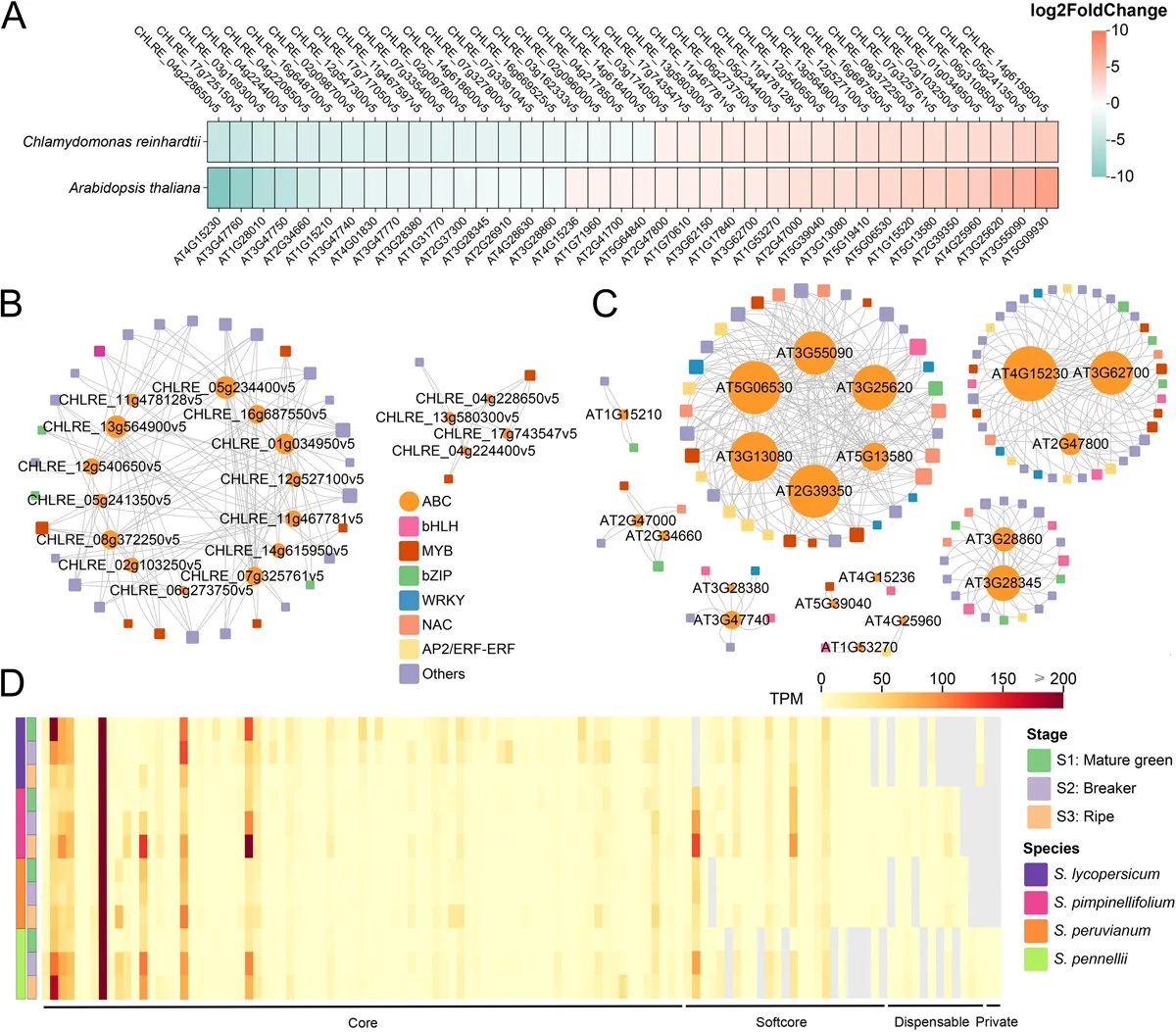
Expression patterns of ABC genes across plant lineages. (A) The differential expression fold changes of ABC genes in *C. reinhardtii* and *A. thaliana* under salt stress. (B) Co-expression network of differentially expressed ABC genes and transcription factors in *C. reinhardtii*. (C) Co-expression network of differentially expressed ABC genes and transcription factors in *A. thaliana*. The sizes of circles and squares represent the connectivity degree. (D) Expression of tomato pan-ABC family in mature green, breaker, and ripening stages of fruit development.

Co-expression network analysis revealed global regulatory differences in ABC genes and transcription factor between *C. reinhardtii* and *A. thaliana* (Fig. 9B and C). *Chlamydomonas reinhardtii* possessed two co-expression modules containing 18 ABC genes, whereas *A. thaliana* had nine co-expression modules containing 20 ABC genes. Compared with *C. reinhardtii*, three co-expression modules in *A. thaliana* exhibited greater connectivity.

To investigate the potential role of the ABC gene family in tomato fruit development, we comprehensively analyzed the expression patterns of each ABC OGG across three developmental stages (mature green, breaker, and ripe) in four tomato accessions (*S. lycopersicum*, *S. pimpinellifolium*, *S. peruvianum*, and *S. pennellii*). Overall, 99 ABC OGGs exhibited transcript abundance with transcripts per million (TPM) values exceeding 50, while 34 ABC OGGs had TPM values over 100. Compared to softcore, dispensable, and private ABC OGGs, core ABC OGGs displayed higher expression levels, particularly OG0000002, OG0000008, OG0000019, and OG0000029 (Fig. 9D).

### Plant ABC gene family database

To facilitate community-wide research on plant ABC transporters, we constructed the Plant ABC gene family PlantABC database (http://biodb.com.cn/PlantABC/index.html). This resource integrates comprehensive data for ABC gene family identified from 1108 plant species, systematically classified into eight major evolutionary lineages. The database features an intuitive interface with several functional modules: Home, Statistics, Search, Tools, Download, and Help pages (Fig. 10). Users can efficiently retrieve specific ABC gene families and access detailed annotations. The platform also enables visually comparative analysis of ABC gene characteristic across different plant lineages. All data are freely accessible and available for bulk download, providing a valuable resource for evolutionary studies, functional genomics, and crop improvement research.

**Figure 10:**
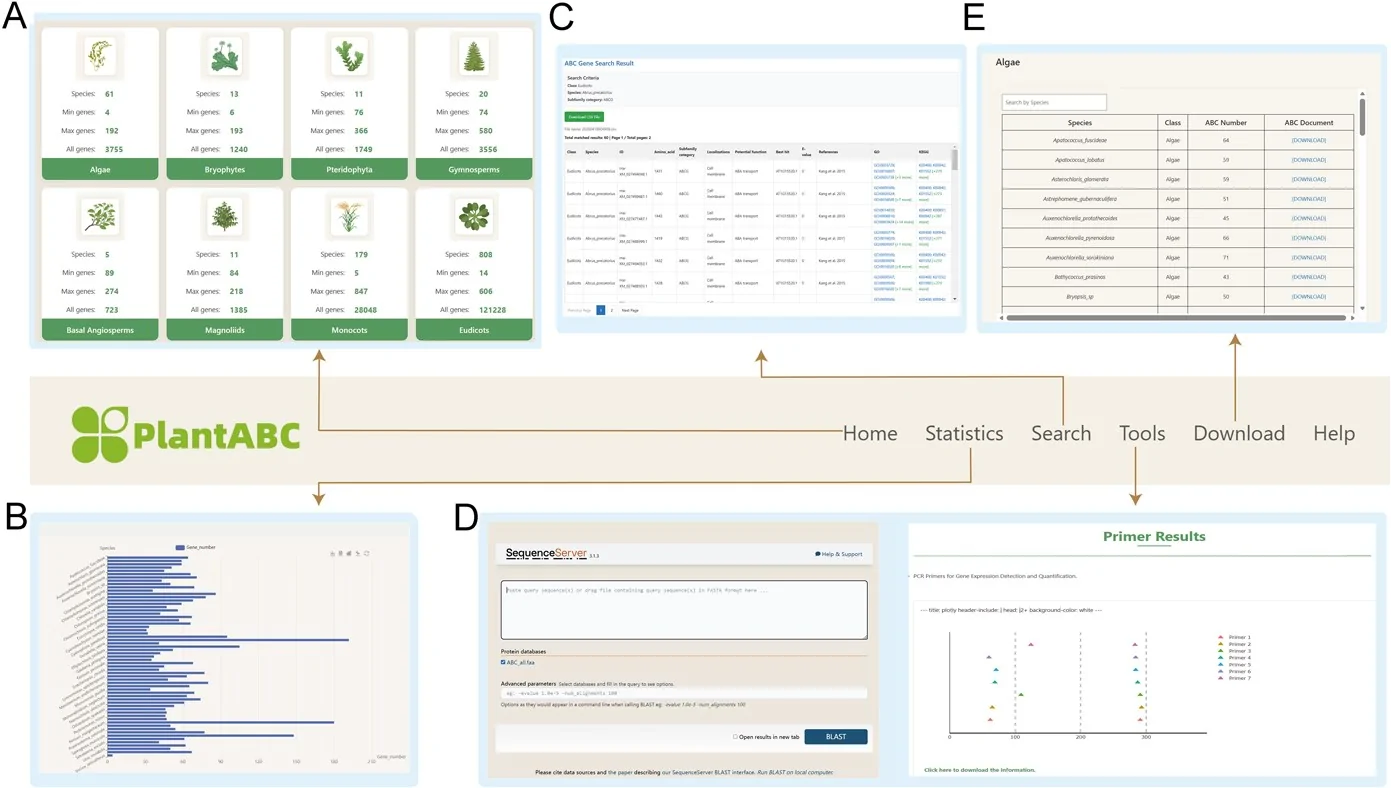
The framework of the PlantABC database modules, including the Home, Statistics, Search, Tools, Download, and Help modules. (A) Home page. (B) Visualization of the quantitative relationship of ABC genes across different species. (C) Result display of the search interface. (D) Tools for BLAST and primer design. (E) Download function for ABC genes of each species.

## Discussion

In this study, the ABC gene family from 1108 plant species, including many horticultural plants and economically important crops, was comprehensively identified and characterized. Based on comparative genomics, the evolution and function of the ABC gene family were systematically analyzed, including comparisons across different plant lineages, phylogenetic relationships, gene duplication and loss, duplication types, collinearity, selective pressure, full-length transposon insertions, pan-family analysis, and protein functional investigations. Additionally, the plant ABC gene family database was established.

A total of 159 077 ABC genes were identified across more than a thousand plant species. Among ABC proteins longer than 1000 amino acids, a distinct bimodal distribution of protein length was observed in monocots and eudicots. This suggests that the functional divergence of longer ABC proteins may have occurred in more advanced angiosperms and potentially contributed to the ecological adaptation of flowering plants. *Arabidopsis* ABCG13 was highly expressed in petals and carpels and was essential for the transport of flower cuticular lipids [38]. In rice, OsABCG3 played a crucial role in pollen development [39]. In *Stenocereus thurberi*, *StABCG11* could cooperate with *StCYP77A* and *StGDSL1* to regulate the synthesis, polymerization, and transport of cuticle components during the early and ripe stages of fruit development [40]. The longer proteins were found in bryophytes, lycopodiophyta, and pteridophyta, and a greater number of ABC gene family members were revealed in other plant groups, suggesting that the ABC gene duplication may play an important role in the process of plant terrestrialization [41, 42].

Phylogenetic analysis based on representative species and a comparison of gene numbers across different lineages in large-scale species indicate that members of the ABCB, ABCC, and ABCG subfamilies have undergone significant expansion in plants. A total of 100 ancestral genes were duplicated in the common ancestor lineage of the nine representative plants, with no gene loss observed. The increase in duplicated genes may have facilitated environmental adaptation through functional divergence after duplication [43]. The *Arabidopsis* ABCG14 gene expanded in tomato, forming two orthologous gene pairs with Solyc08g075430.3 and Solyc08g005580.3. *Arabidopsis* ABCG14 had been confirmed as a transporter protein responsible for the transport of cytokinins from the roots to the stems [44, 45]. The Ka/Ks analysis of the orthologous gene pairs between tomato, *Arabidopsis*, and rice indicated that all subfamily genes underwent strong purifying selection during evolution. In the ABCB and ABCG subfamilies, significant differences in Ka/Ks values among some genes suggested the important role of these subfamilies in adapting to variable environments [46, 47]. Transposons, as active genomic elements, facilitated the adaptive evolution of land plants [48]. Various types of transposons, including long terminal repeat retrotransposons (LTRs), were found to insert into the gene bodies and flanking regions of ABC genes in tomato, *Arabidopsis*, and rice, suggesting that transposable elements may have facilitated the expansion of the ABC gene family in higher angiosperms and potentially contributed to the emergence of new gene functions.

A total of 1548 tomato ABC genes were identified at the pan-genome level in 12 tomato accessions and were classified into 79 core ABC OGGs, 24 softcore ABC OGGs, 13 dispensable ABC OGGs, and 7 private ABC OGGs. The ABC genes of *S. lycopersicum* were found only in 107 homologous groups. This indicated that pan-genome-based ABC family analysis overcame the limitations of single cultivar genome analysis and more comprehensively captured the diversity of tomato ABC genes [49, 50]. Some studies also confirmed the unique advantages of pan-gene family analysis [51–53]. Copy number variation was found between different tomato accessions, suggesting that these genes underwent different evolutionary selections across accessions. Ka/Ks analysis showed that core ABC OGGs experienced the strongest purifying selection, likely involved in basic physiological processes. Dispensable ABC OGGs exhibited more relaxed purifying selection, highlighting their potential for diverse biological functions, possibly related to adaptive evolution.

Plant gene function research has been greatly advanced by specialized functional gene databases [54–57], and a systematic plant ABC genes database is urgently required. The PlantABC database was established based on the data from this study. The 159077ABC genes identified in this study have been comprehensively annotated for function based on the well-characterized *Arabidopsis* ABC genes. All information is available for query in the database. The species in the database included important food crops, vegetables, fruits, and their closely related species, which will greatly facilitate research on the association between ABC genes and key agronomic traits such as yield and quality. In the future, we will annually update information and enrich function of PlantABC database, providing a user-friendly and comprehensive resource platform for studies of ABC gene family.

## Materials and methods

### Data retrieval

Genomic data of 1108 plant species were collected from TAIR [58], NCBI [59], PlantGIR [36], and other databases (Table S1). The species encompassed whole plant lineages, including 61 algae species, 13 bryophytes species, 11 pteridophyta species, 20 gymnosperms species, 5 basal angiosperms species, 11 magnoliids species, 179 monocots species, and 808 eudicots species.

### Identification of ABC gene family

The hidden Markov model (HMM) file (PF00005) for the conserved domain of ABC proteins was obtained from the InterPro database [60]. The ABC proteins were identified by HMMER v3.4 with E-value cutoff of <1 × 10^−5^ [61]. Candidate protein sequences with full-length > 180 aa and NBD core domains >120 aa were further screened as final ABC family members. The function annotations of all ABC genes were performed using the GFAP tool [62].

### Comparative analysis of ABC gene family

All the ABC protein sequences were subjected to BLASTP using DIAMOND v2.1.9.163, with *A. thaliana* ABC proteins as the database [63]. The subfamily category and subcellular localization of each ABC protein were considered to be the same as those of the best-hit *A. thaliana* ABC protein. The number and protein lengths of ABC members between subfamily in different plant lineages were analyzed and compared.

### Phylogenetic tree construction

Nine species were selected as representative species for different plant lineages, including *C. reinhardtii* (algae), *P. patens* (bryophytes), *S. moellendorffii* (pteridophyta), *Picea abies* (gymnosperms), *Amborella trichopoda* (basal angiosperms), *Aristolochia fimbriata* (magnoliids), *Oryza sativa* (monocots), *A. thaliana* (eudicots), and *S. lycopersicum* (eudicots). These species hold significant evolutionary importance and representativeness. The ABC protein sequences of the nine species were aligned using MAFFT v7.525 (--auto) [64], followed by trimming with trimAl v1.5.0 (automated1) [65]. The phylogenetic tree was constructed using FastTree v2.1.11, with default parameters (JTT model) [66]. The phylogenetic tree was visualized using iTOL [67].

### Conserved motif identification and distribution analysis

The conserved motifs of ABC proteins in nine representative plants were detected using MEME (https://meme-suite.org/meme/tools/meme) [68]. The distribution differences were analyzed across nine species. The motif frequency of all proteins was displayed using Chiplot [69].

### Duplication and loss inference of ABC gene family

The duplication and loss of ABC family genes were analyzed based on the phylogenetic comparison between the gene tree and true species tree using Notung v2.9.1.5 [70].

### Collinearity analysis and duplication type detection

The synteny of ABC gene family within and between species was detected using MCScanX [71]. Protein sequences were aligned using the blastp program in diamond v2.1.9.163, with an *e*-value of 1 × 10^−3^. Synteny blocks were detected using MCScanX with default parameters. The duplication types of genes were detected using the duplicate_gene_classifier program in MCScanX software.

### Selective pressure analysis

Intra-species synteny of ABC genes from MCScanX was displayed using the Advanced Circos module in TBtools [72]. The synteny of ABC genes among the three species from MCScanX was visualized using synvisio (https://synvisio.github.io/#/). The Ka, Ks, and Ka/Ks values of orthologous gene pairs between *A. thaliana, O. sativa,* and *S. lycopersicum* were analyzed using KaKs_Calculator 2.0 [73] and ParaAT 2.0 [74].

### Full-length transposable element analysis

The full-length TEs of representative species genomes were detected using HiTE v3.3.2 [75], including all types of retrotransposons and DNA transposons. The genomic coordinates of the gene body, upstream 2000 bp, and downstream 2000 bp of the ABC genes were extracted and calculated based on genomic annotation information. The transposon was considered to be inserted into the ABC feature region only when its sequences overlap with >20 bp of the ABC feature sequences.

### Identification of tomato Pan-ABC gene family

Genomic data from 12 tomato accessions were retrieved from the CaasTomato database (http://caastomato.biocloud.net/home), including 1 cultivated species, 1 cultivated variety, and 10 wild species (Table S2) [76]. The ABC proteins of each tomato accession were identified using HMMER v3.4.

### Orthologous gene group analysis

The OGGs of all tomato ABC genes were constructed using orthofinder v2.5.5 with default parameters. The clustered OGGs were classified into four categories: the core OGGs shared by all 12 tomato accessions, the softcore OGGs containing ABC genes that presented in 11 accessions, the dispensable OGGs accommodating ABC genes that presented in 2–10 accessions, the private OGGs containing ABC genes that only existed in 1 accession.

### Selective pressure and Gene Ontology enrichment analysis

The Ka, Ks, and Ka/Ks values of gene pairs in each OGG were calculated using ParaAT 2.0 and KaKs_Calculator 2.0 with the default model (Model Averaging, MA). The functional annotation of all genomic genes was conducted using eggNOG-mapper (http://eggnog-mapper.embl.de/) [77], and Gene Ontology (GO) and Kyoto Encyclopedia of Genes and Genomes (KEGG) annotation were further cleaned. The GO enrichment analyses were performed using the clusterProfiler 4.6.0 R package with the BH-FDR multiple testing correction method.

### Expression pattern analysis

The transcriptome data were obtained from the SRA database [78], including: (i) *C. reinhardtii* (SRP341124) and *A. thaliana* (SRP517608) under salt stress and (ii) fruit tissue (SRP361039) at the mature green, breaker, and ripe stages in *S. lycopersicum*, *S. pimpinellifolium*, *S. peruvianum*, and *S. pennellii*. The raw sequencing data were assessed for quality through FastQC v0.12.1. Adapter sequences were removed and the first 12 bases of reads were trimmed to obtain clean reads using Trimmomatic v0.39 [79]. The genome index was constructed, and clean reads were mapped to the reference genome by HISAT2 v2.2.1 [80]. SAM files were then converted to reordered BAM files via SAMtools v1.7 [81]. Transcript abundances were estimated and TPM values were calculated using StringTie v2.1.7 [82]. The count values were derived using the prepDE.py3 program provided by StringTie v2.1.7 [82]. The DEGs were analyzed with DESeq2 [83]. The screening criteria for DEGs were |log2FoldChange| > 1 and padj <0.05. Gene co-expression analysis was performed using the Pearson method, with thresholds set at correlation < −0.8 and *P*-value <1 × 10^−5^. The co-expression results were visualized using the Cytoscape v3.9.1 [84].

### Database construction

The PlantABC database was developed under the Linux system, aiming to provide online search and browsing capabilities through a user-friendly web interface. The web interface was constructed using HTML, CSS, and JavaScript, with MySQL employed for database management. Data visualization was implemented using R, and the system runs on an Apache server.

## Supplementary Material

Web_Material_uhag209

## Data Availability

All data generated and analyzed during this study are included in this article and supplementary file.
