# Supplementary material for: Large-scale identification and evolutionary analysis of ATP-binding cassette transporters in cross-lineage plant species
Source: Hortic Res. 2026 Jun 1;13(11):uhag209. doi: 10.1093/hr/uhag209 (PMC13711656; doi:10.1093/hr/uhag209)
Supplement: Supplementary file 1 [file web_material_uhag209.zip › Supplementary Figures.pdf]

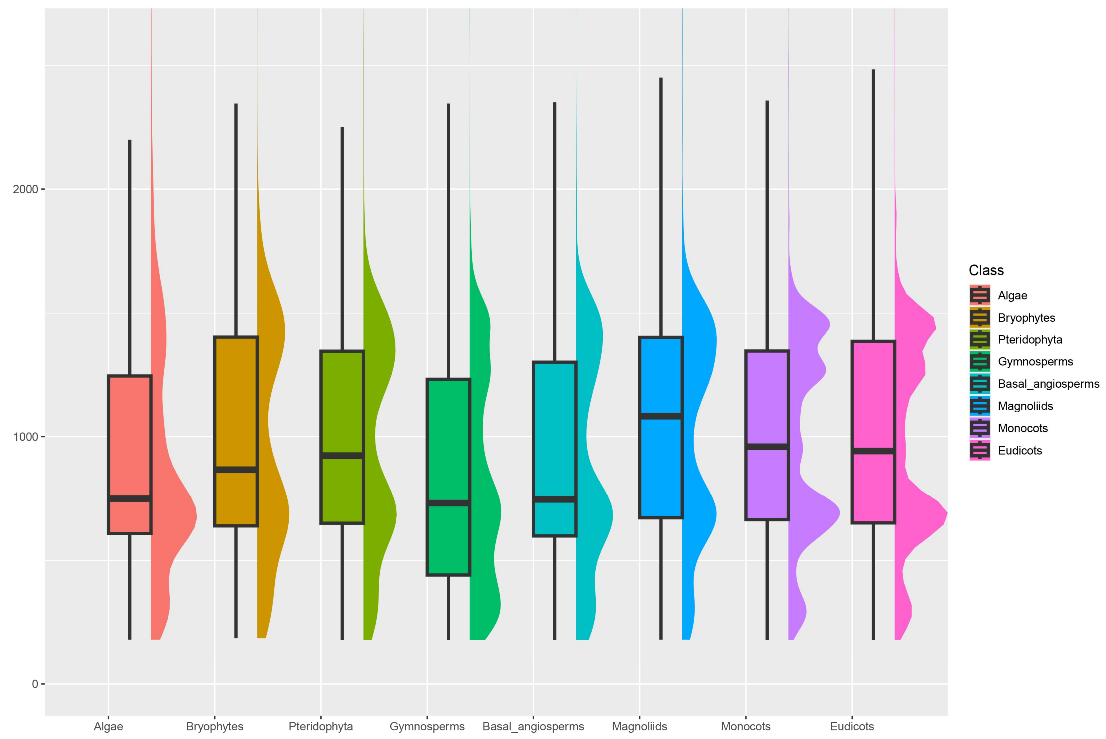

Figure S1 The amino acid sequence length of ABC family proteins in eight lineages, with different colors representing different lineage.

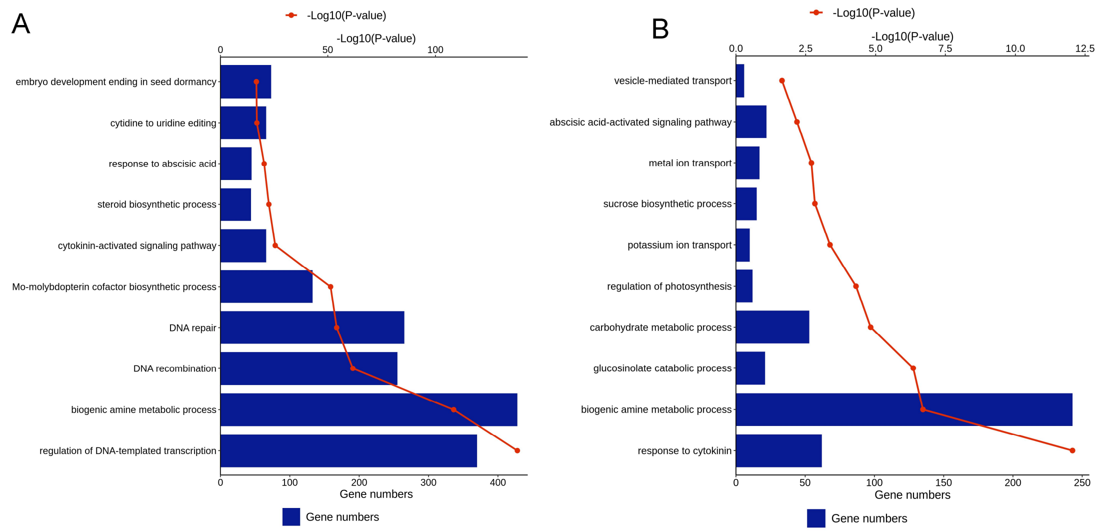

Figure S2 GO enrichment of ABC proteins with length exceeding 1000 amino acids in Eudicots. (**A**) GO term of the proteins contained in the above peak. (**B**) GO term of the proteins contained in the below peak.

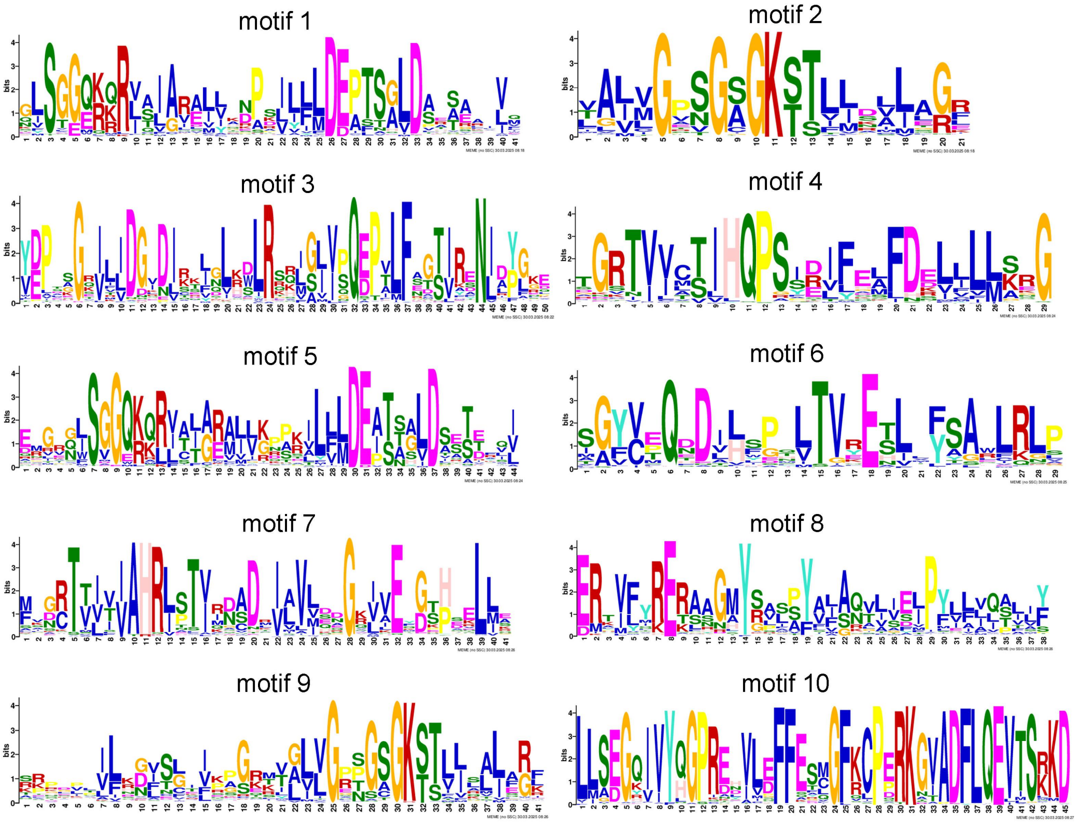

Figure S3 Conservative motif sequence of representative species. The size of the grapheme represented the conservation of the amino acid at that position.
